# Intracellular Ca^2+^ Increases and Connexin 43 Hemichannel Opening Are Necessary but Not Sufficient for Thy-1-Induced Astrocyte Migration

**DOI:** 10.3390/ijms19082179

**Published:** 2018-07-26

**Authors:** Raúl Lagos-Cabré, Marianne Brenet, Jorge Díaz, Ramón D. Pérez, Leonardo A. Pérez, Rodrigo Herrera-Molina, Andrew F. G. Quest, Lisette Leyton

**Affiliations:** 1 Cellular Communication Laboratory, Programa de Biología Celular y Molecular, Instituto de Ciencias Biomédicas (ICBM), Facultad de Medicina, Universidad de Chile, Santiago 838-0453, Chile; rclagos@uc.cl (R.L.-C.); mbrenetrivas@gmail.com (M.B.); jbdiaz1@gmail.com (J.D.); ramonbq@gmail.com (R.D.P.); leonardo.perez@ug.uchile.cl (L.A.P.); aquest@med.uchile.cl (A.F.G.Q.); 2 Advanced Center for Chronic Diseases (ACCDiS), Center for Studies on Exercise, Metabolism and Cancer (CEMC), Instituto de Ciencias Biomédicas (ICBM), Facultad de Medicina, Universidad de Chile, Santiago 838-0453, Chile; 3 Leibniz Institute for Neurobiology, 39118 Magdeburg, Germany; rherrera@lin-magdeburg.de; 4 Centro Integrativo de Biología y Química Aplicada, Universidad Bernardo O’Higgins, Santiago 837-0993, Chile

**Keywords:** connexin 43 hemichannels, gap junctions, cell polarization, cell migration, inflammation, reactive astrocytes, neuronal signals

## Abstract

Under pro-inflammatory conditions, astrocytes become reactive and acquire a migratory phenotype. Our results show that hemichannels formed by connexin 43 (Cx43) play an important role in Thy-1-induced astrocyte migration. The neuronal protein Thy-1 binds to αvβ3 integrin in astrocytes, thereby leading to intricate signaling pathways that include calcium (Ca^2+^) release from intracellular stores, opening of Cx43 hemichannels, release of ATP, activation of P2X7 receptor, and Ca^2+^ influx. However, because these Thy-1 effects occur exclusively in reactive astrocytes, we wondered whether by elevating calcium levels and promoting hemichannel opening we could prompt non-reactive astrocytes to respond to Thy-1. Cx43 immunoreactivity increased at juxta-membrane sites, where hemichannels (not gap junctions) participate in astrocyte polarization and migration stimulated by Thy-1. Also, intracellular Ca^2+^ increase, due to ionomycin treatment, induced hemichannel opening, but activated astrocyte migration only partially, and this limitation was overcome by pre-treatment with tumor necrosis factor (TNF) and Thy-1. Finally, αvβ3 integrin formed membrane clusters after TNF stimulation or overexpression of β3 integrin. We suggest that these microclusters are required for cells to respond to Thy-1 stimulation. Therefore, the large increase in intracellular Ca^2+^ and hemichannel opening induced by ionomycin are required, but not sufficient, to permit Thy-1-induced astrocyte migration. Thus, we suggest that proinflammatory stimuli prompt astrocytes to respond to migratory signals of neuronal cells.

## 1. Introduction

Astrocytes are the most abundant cell type in the central nervous system (CNS); however, their function has always been overshadowed by neurons. Astrocytes not only help and nourish neurons, but also participate in synapse function [1]. In addition, they represent the first line of defense after injury by taking up neurotransmitters and ions present at toxic levels, enclosing the lesion, avoiding the propagation of damaging molecules, repairing the blood brain barrier, and forming the glial scar [2,3]. To achieve all of these functions, communication between astrocytes and other surrounding cells, such as neurons, is essential.

We have previously provided evidence for the existence of an intricate molecular mechanism in astrocytes triggered by the neuronal surface molecule Thy-1, which may explain the lack of axonal repair upon central nervous system (CNS) injury in an astrocyte-dependent manner. Nevertheless, astrocytes first need to become “reactive” and increase the levels of membrane receptors and channels in order to respond to Thy-1 [4,5]. Reactive astrocytes are characterized by morphological changes (i.e., increase in size and arborization of processes) and substantial changes in the protein expression profile as a response to pathological conditions in the CNS, such as neurodegenerative diseases, stroke or traumatic injuries [2,3,4,5]. The “reactive” phenotype can also be replicated in vitro by the addition of proinflammatory cytokines, such as tumor necrosis factor TNF or IL-1β [4], which are released by reactive astrocytes and microglia in an injured brain [6]. These reactive astrocytes increase the expression of the two reported Thy-1 receptors, αvβ3 integrin and Syndecan-4, among other proteins [4]. When bound to Thy-1, the engaged receptors trigger an intracellular signaling cascade involving FAK, PLCγ, inositol 1,4,5-trisphosphate receptor (IP_3_R) activation, Ca^2+^ release from the endoplasmic reticulum, connexin (Cx)- and pannexin (Px)-hemichannel opening, ATP release, and finally P2X7 receptor (P2X7R) activation, which further increases intracellular Ca^2+^ concentrations ([Ca^2+^]i) [4,5,6,7,8]. In this complex sequence, connexin 43 (Cx43) channels play an important role not only to allow the release of ATP to the extracellular medium, but also to communicate with the surrounding cells by gap junctions [5,9,10].

After inducing astrocyte reactivity in vitro, Cx43 is overexpressed and pharmacological inhibition precludes astrocyte migration [4,5,11]. In normal conditions, Cx43 is mostly localized in intracellular vesicles. But induction of astrocyte reactivity with TNF triggers Cx43 relocalization to a juxta-membrane zone in primary astrocytes [4]. Cx43 is the most abundantly expressed Cx family member in the CNS and is involved in cell-cell communication by forming gap junctions between astrocytes or astrocytes and neurons [10,12]. In a physiological setting, Cx43-mediated astrocyte-neuron gap junctions control the delivery of energy metabolites from astrocytes to wake-promoting orexin neurons, thus regulating the wake-sleep cycle in mice [12]. However, in a proinflammatory state, such as that induced by conditioned media from microglia, astrocytes increase their permeability by opening Cx43 hemichannels, and decrease Cx43 gap junction communication [13]. Moreover, pro-inflammatory conditions induced by cytokines or neurodegenerative diseases also increase Cx43 protein levels and hemichannel activity [4,14], suggesting that under these conditions, gap junction activity is diminished, while hemichannels appear more active. Furthermore, the role of Cx43 in hemichannel formation and astrocyte migration appears especially relevant in brain injuries, since specific hemichannel inhibitory peptides abolish astrocyte migration [5], an important process required for glial scar formation [15].

The increased levels of Cx43 during astrocyte reactivity help maintain the reactive phenotype of astrocytes and microglia by the release of molecules, such as ATP and glutamate [3,16]. In this context, it is worth noting that reactive astrocytes derived from a mouse model for the neurodegenerative disease Amyotrophic Lateral Sclerosis (ALS) express high levels of Cx43 that help sustain increased [Ca^2+^]i induced by mechanical stimulation or by ATP, effects that are inhibited by a Cx43-blocking peptide [14]. The increase in Ca^2+^ signals are, in turn, important for hemichannel opening, and enhanced [Ca^2+^]i provoked by ionophores induces hemichannel opening in astroglioma cells [9,17], as well as astrocytoma migration [17]; however, the effect of ionophore treatment in non-reactive or reactive astrocytes remained unknown. Therefore, we tested the hypothesis that an increase in [Ca^2+^]i levels and an induction of the hemichannel opening prompt non-reactive astrocytes to respond to Thy-1.

Here, we show that Thy-1 enhanced cell surface Cx43 levels and induced hemichannel opening resulting in cell polarization. Additionally, TNF treatment or enhanced surface levels of αvβ3 integrin by β3 integrin overexpression promotes β3 integrin microcluster formation, clusters that are suggested here to be necessary for astrocytes to respond to neuronal migratory signals. Moreover, we found that ionomycin induces the hemichannel opening independently of the reactive phenotype of astrocytes, an effect that is potentiated by Thy-1. Ionomycin also promotes astrocyte migration; however, ionomycin and/or Thy-1 require TNF for effective cell migration.

## 2. Results

### 2.1. Thy-1 Enhances Cell Surface Cx43 Levels, Opens Hemichannels, and Induces Cell Polarization

**Figure 1:**
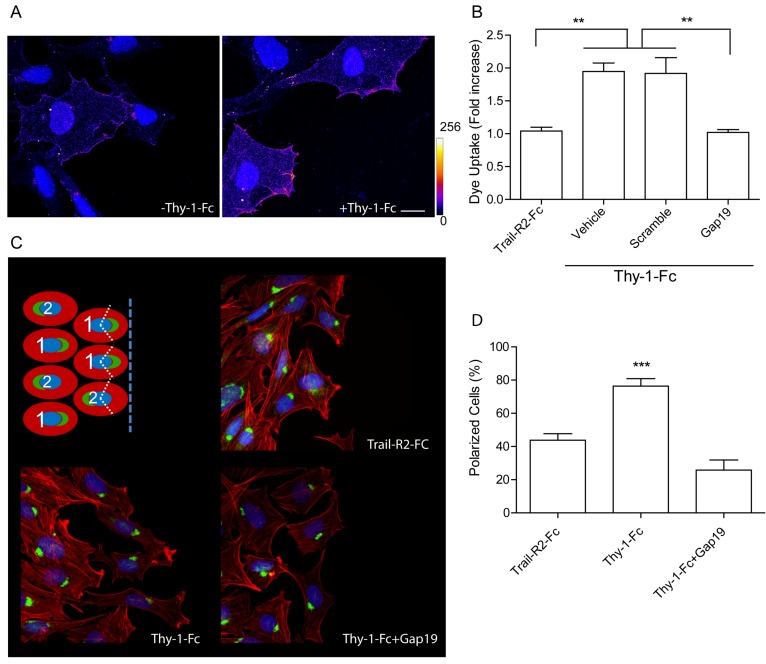
Thy-1 enhances cell surface connexin 43 (Cx43) levels, opens hemichannels, and induces cell polarization. (**A**) Representative images of Cx43 localization in DITNC1 astrocytes before and after Thy-1 addition for 10 min. The heat map of fluorescence intensity indicates relative levels of Cx43, going from 0 (blue) to 256 (white). Scale bar = 10 µm; (**B**) Lucifer Yellow uptake quantification in astrocytes treated with tumor necrosis factor (TNF) for 48h prior to stimulation with Thy-1-Fc or Trail-R2-Fc (used as a negative control) [4,5]). Cells were pre-incubated with vehicle, a scrambled peptide, or Gap19; (**C**) Representative images of cell polarization. Confluent cultures of astrocytes were scratched with a pipette tip and Golgi localization was determined using an anti-giantin antibody. Phalloidin-rhodamine was used to identify the cell border. Polarization was defined as positioning of the Golgi apparatus within an angle of 120° facing the wounded area (see scheme in the top left panel). Scale bar = 10 µm; (**D**) Quantification of Figure 1C. Results from at least three independent experiments showing mean ± s.e.m. ** *p* < 0.01, *** *p* < 0.001.

Our previously reported evidence indicated that Thy-1 stimulation induces the release of ATP to the extracellular medium through Cx43 and Px1 hemichannels, thereby activating P2X7R and Ca^2+^ entry, which are ultimately required for the adhesion, polarization, and migration of astrocyte cell lines, as well as primary rat neonatal astrocytes in culture [4,5]. We established the importance of the Cx43 hemichannel opening by incubating astrocytes with Heptanol, a rather unspecific Cx inhibitor [5], and with more specific Cx inhibitors, such as the Gap26 and Gap27 peptides, which block both Cx43 hemichannels and gap junctions [5]. Thus, we now used different tools to completely rule out a possible involvement of gap junctions in the cellular responses. First, we assessed Cx43 expression by indirect immunofluorescence in isolated DITNC1 astrocytes. In these cells, which behave as reactive astrocytes, surface staining for Cx43 was detected in the plasma membrane of isolated cells, indicating that Cx43 was present in the form of hemichannels (Figure 1A). Interestingly, when cells were treated with Thy-1 for 10 min, we observed that juxta-membrane Cx43 levels increased, compared to control conditions (Figure 1A), suggesting a redistribution of this protein in Thy-1-treated cells. We then measured the opening of Cx43 hemichannels by monitoring Lucifer Yellow (LY) uptake. Increased LY uptake was observed after Thy-1 addition, which was precluded when cells were preincubated with Gap19 (Figure 1B), a peptide that exclusively blocks Cx43 hemichannels [5]. By using the scrambled peptide as a control for Gap19, we found mean average values that were similar to those obtained when only Thy-1 was added in serum-free medium (Figure 1B). TRAIL-R2-Fc is a used negative control in studies with the fusion protein Thy-1-Fc (Figure 1B). We have previously shown that TRAIL-R2-Fc does not bind to cell surface receptors and does not elicit cellular responses [4,5]. Considering that cell polarization was assessed in experiments employing confluent cell monolayers, where gap junctions could form at the cell-cell contact sites, we tested whether cell polarization was affected by the inhibition of Cx43 in the form of hemichannels. To this end, DITNC1 astrocyte monolayers were scratched with a pipette tip and then stimulated with Thy-1, and cell polarization was measured by evaluating the Golgi apparatus reorientation towards the wounded site [5]. Inhibition of Cx43 with Gap19 completely blocked cell polarization as shown (Figure 1C) and quantified (Figure 1D). Taken together, these results demonstrate that the opening of Cx43 hemichannels is triggered by the addition of Thy-1 and that these hemichannels, rather than gap junctions, are involved in cell polarization. Moreover, the results indicate that stimulation with Thy-1 elevates Cx43 levels at the cell surface.

### 2.2. Levels of αvβ3 Integrin Increase at the Cell Membrane and Form β3 Integrin Receptor Microclusters

**Figure 2:**
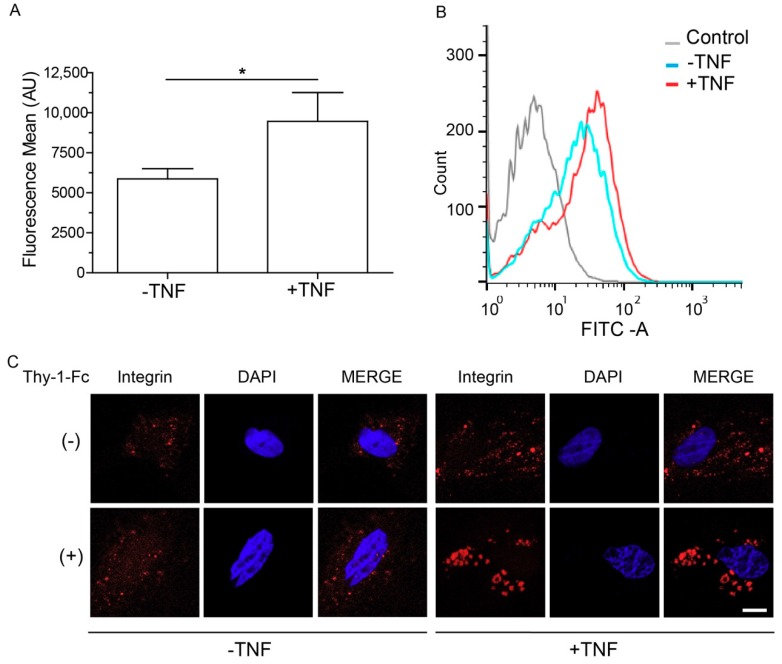
Expression levels of β3 integrin increase at the cell surface and form β3 integrin receptor clusters. (**A**) Analysis of β3 integrin abundance at the cell surface by flow cytometry (FACS) analysis performed in non-permeabilized primary astrocytes. The graph depicts the mean fluorescence intensity in astrocytes in the absence and the presence of TNF. Values in the graph are mean ± s.e.m. of five independent experiments per condition. * *p* < 0.05; (**B**) The histogram shows a comparison of β3 integrin expression levels in cells that were within the gates of the control cells, cells without TNF or with TNF, from one representative experiment. Control condition was carried out in the absence of the primary antibody (gray line). Results correspond to 10,000 events per sample; (**C**) Confocal images of non-treated primary astrocytes or cells treated with TNF (48 h) in the presence or absence of Thy-1 (15 min). Non-permeabilized cells were stained for αvβ3 integrin (red) and DAPI (blue), which was used to stain the nuclei. The merge of both colors is also shown. Scale bar = 5 µm.

αvβ3 integrin is a receptor for Thy-1 in astrocytes, and β3 integrin protein levels reportedly increase in whole cell lysates after incubation with TNF [4]. We therefore analyzed β3 integrin levels by cell cytometry to test the surface localization of this protein. We found that compared to non-treated samples, β3 integrin levels were elevated at the cell surface in the presence of TNF (Figure 2A,B). Despite the fact that TNF treatment increases the expression of various receptors, it does not affect Ca^2+^ levels nor cell migration, unless Thy-1 is added [4]. It is known that effective receptor-triggered intracellular signals are regulated by the interactions of proteins at the plasma membrane and that such interactions depend on their concentration, spatial localization, and ability to form complexes and clusters [18]. We therefore explored the possibility that TNF prompts cells to respond to Thy-1 by favoring receptor cluster formation upon increasing the amount of β3 integrin at the plasma membrane. We assessed the spatial localization of β3 integrin in non-permeabilized astrocytes treated with TNF by confocal microscopy. Clustering of β3 integrin at the surface was only observed in primary astrocytes treated with TNF (Figure 2C) and, interestingly, Thy-1 elevated the number and size of the microclusters only in TNF-treated astrocytes (Figure 2C). The results suggest that TNF prepares cells to respond more efficiently to Thy-1-induced cellular signaling and favors the migratory phenotype by promoting the formation of β3 integrin receptor microclusters.

### 2.3. β3 Integrin Overexpression Leads to Formation of Integrin Microclusters at the Cell Surface

**Figure 3:**
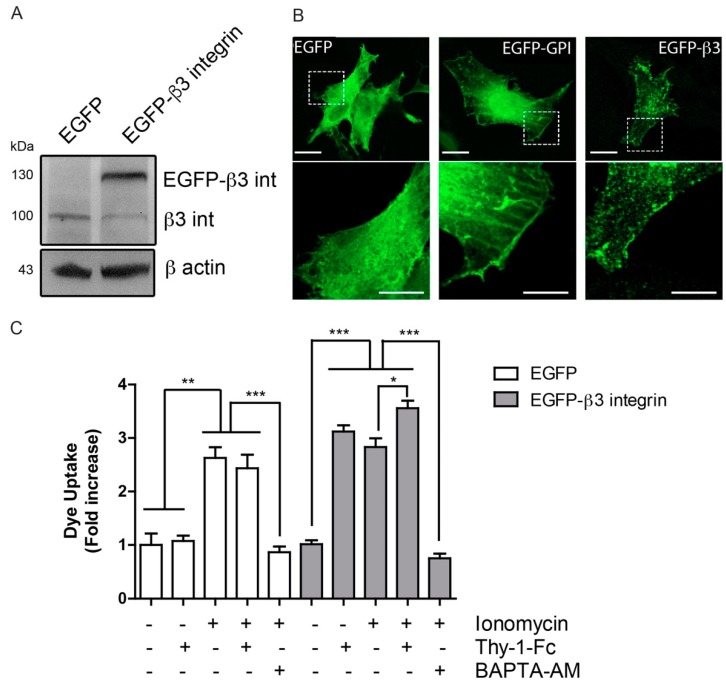
β3 integrin overexpression forms integrin clusters at the cell surface. (**A**) Cell lysates of primary astrocytes transfected with pEGFP or pEGFP-β3 integrin were immunoblotted for β3 integrin and β-actin as a loading control; (**B**) Confocal images of primary astrocytes transfected with pEGFP, pEGFP-GPI or pEGFP-β3 integrin were acquired 24 h after plasmid transfection. Scale bar = 20 µm. Outlined area defines the optically zoomed region shown below each photograph (bar = 10 µm); (**C**) Dye uptake by primary astrocytes transfected as in A, stimulated with Thy-1-Fc or ionomicyn (1 µM) for 10 min, or treated with the intracellular calcium chelator BAPTA-AM (5 µM) for 30 min prior to stimuli. Values in the graph are the mean fluorescence intensity normalized to the non-stimulated condition. Results show mean ± s.e.m. from three independent experiments, * *p* < 0.05, ** *p* < 0.01, *** *p* < 0.001.

We then reasoned that if TNF increases β3 integrin expression at the glial cell membrane and patch formation (Figure 2), the overexpression of EGFP-β3 integrin in these cells should lead to a similar outcome. To test this, EGFP-β3 integrin expression and cell localization of the tagged protein were monitored by Western blotting and confocal microscopy, respectively. Presence of EGFP-β3 integrin, as well as of endogenous β3 integrin were detected in transfected cells (Figure 3A). Additionally, EGFP-β3 integrin appeared to occasionally form small clusters at the membrane of resting astrocytes (Figure 3B). This clustering was not observed in astrocytes expressing GFP alone. Moreover, and as expected, default localization of GFP-GPI in lipid rafts showed a more disseminated distribution throughout the cell membrane surface, compared to clustered β3 integrin-GFP (Figure 3B). Since our previous data has indicated that cells overexpressing β3 integrin do not develop a migratory phenotype unless Thy-1 is added [4], we propose that β3 integrin overexpression, only predisposes the cells to be more migratory. To test this hypothesis, we performed dye uptake experiments, indicative of hemichannel opening. Ionomycin treatment opened hemichannels, and Thy-1 increased the effect of ionomycin, only when β3 integrin was overexpressed (Figure 3C). Moreover, addition of the Ca^2+^ chelator BAPTA inhibited the ionomycin effect. Considering that Thy-1 induces cell migration of β3 integrin-overexpressing astrocytes [4], the effect of Thy-1 on hemichannel opening was not surprising (Figure 3C). These data indicate that β3 integrin overexpression prompts cells to respond to Thy-1 by generating microclusters of β3 integrin at the cell surface.

### 2.4. Hemichannel Opening and [Ca^2+^]i Increases Are Necessary, but Not Sufficient to Promote Cell Migration

**Figure 4:**
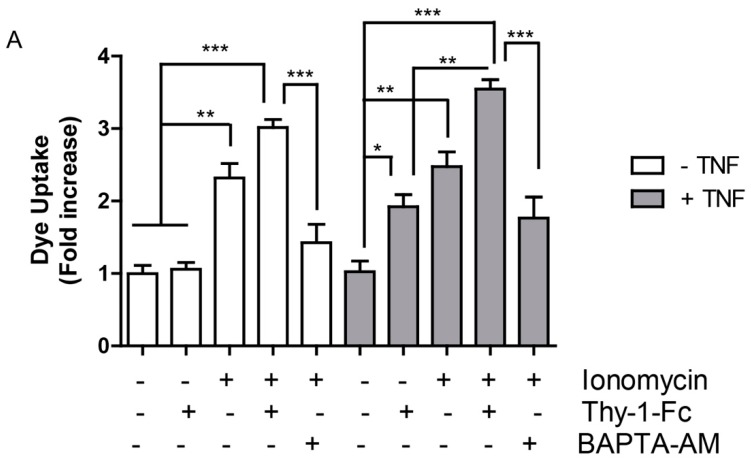

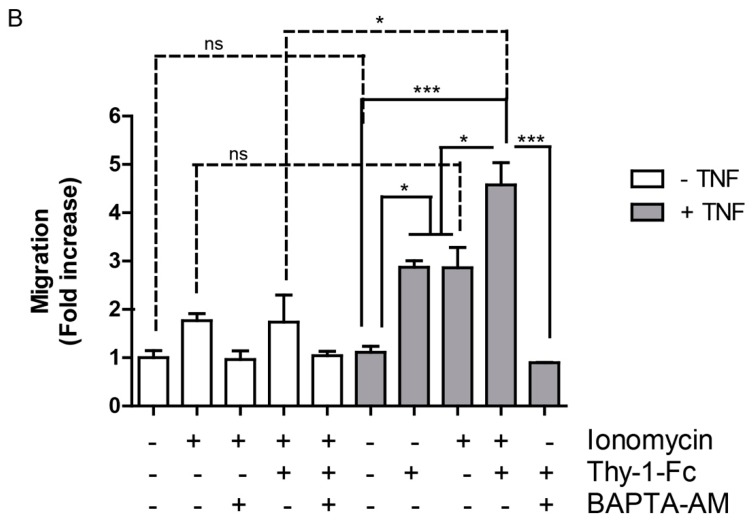
Hemichannel opening and ionomycin-induced intracellular calcium (Ca^2+^) increase are necessary, but not sufficient, to promote Thy-1-induced cell migration. (**A**) Quantification of Lucifer yellow uptake by primary astrocytes pre-incubated with TNF (10 ng/mL for 48 h), BAPTA-AM (5 µM for 30 min) and then stimulated with Thy-1-Fc or ionomicyn (1 µM) for 10 min. Values in the graph correspond to the mean fluorescence intensity normalized to the non-stimulated condition; (**B**) Cell migration quantification of primary astrocytes incubated as indicated in (**A**). Cells were allowed to migrate for 2 h in inserts pre-coated on the lower side with fibronectin (2 μg/mL). Migrating cells were visualized by crystal violet staining of cells on the lower side of the inserts. Data were normalized to non-treated cells and are shown as the average of 7 different fields from each independent experiment, *n* = 3, ns = non-significant, * *p* < 0.05, ** *p* < 0.01, *** *p* < 0.001. Solid lines indicate comparisons of pairs under the same experimental conditions, i.e., −TNF or +TNF. Dashed lines are comparisons of pairs between experimental conditions, i.e., between −TNF and +TNF.

In rat astrocytes, Thy-1-induced migration is a Ca^2+^-dependent event, which can be blocked either by the addition of an IP_3_R blocker, such as 2-APB or by silencing the P2X7R [5]. These results support the notion that increased [Ca^2+^]i due to release from intracellular stores, as well as through Ca^2+^ influx, are necessary for astrocyte migration. The addition of Thy-1 enhances [Ca^2+^]i in astrocytes prior to hemichannel opening and cell migration [4,5]. Therefore, we tested the hypothesis that ionophore-mediated Ca^2+^ increases may lead to hemichannel opening and cell migration. We found that dye uptake was increased in ionomycin-treated cells, whereas TNF had no effect on its own. Pre-treatment of cells with TNF, followed by ionomycin, did not elevate the effect of ionomycin (Figure 4A). Considering that Thy-1 without TNF-pretreatment had no effect on hemichannel opening, an unexpected result was that Thy-1 synergized with ionomycin, even in the absence of TNF (Figure 4A). On the other hand, BAPTA-AM inhibited both the effect of ionomycin and that induced by Thy-1. Furthermore, transmigration, tested in a Boyden chamber assay, was only partially activated by the Ca^2+^ ionophore and intriguingly, required TNF-pretreatment to efficiently trigger this process (Figure 4B). Moreover, Thy-1, along with ionomycin, did not increase cell migration induced only by ionomycin, except when added to TNF-treated cells (Figure 4B). These results support the notion that neither hemichannel opening nor the elevation of [Ca^2+^]i is sufficient for Thy-1-induced astrocyte migration.

## 3. Discussion

**Figure 5:**
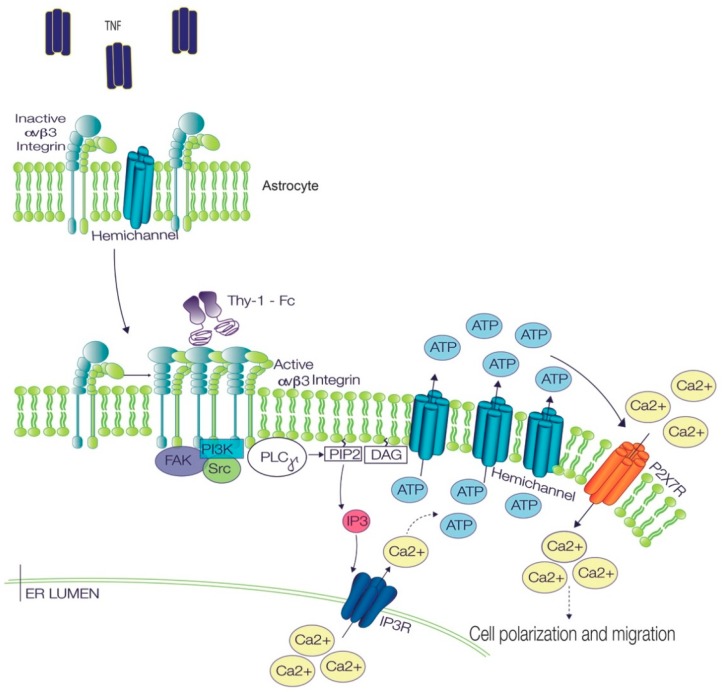
Signaling pathways involved in TNF/Thy-1-induced astrocyte polarization and migration. In astrocytes, TNF treatment induces integrin microcluster formation, increases Cx43 levels at the plasma membrane and prepares cells to respond to Thy-1. Once Thy-1 binds to the integrin microclusters, the receptors oligomerize further, thereby leading to the formation of bigger clusters and integrin activation. The active integrin protein then recruits signaling molecules, such as FAK, PI3K, Src, PLCγ. The activation of the phospholipase generates diacylglycerol (DAG) and IP_3_, which activates the inositol 1,4,5-trisphosphate receptor (IP_3_R) to allow the release of Ca^2+^ from intracellular stores. Increased intracellular Ca^2+^ leads to the opening of hemichannels, the consequent release of ATP to the extracellular medium, and the activation of P2X7 receptors, which allow Ca^2+^ entry into the cell. This signaling pathway is part of a more complex chain of events that eventually leads to cell polarization and migration (modified from [5]). Arrows with solid lines indicate proven connections which may or may not be direct. Arrows with dashed lines indicate connections with non-specified/multiple in between steps.

In this study, we show that Ca^2+^ signals and Cx43 hemichannel opening participate as key steps in the regulation of Thy-1-induced astrocyte migration. Despite their crucial role, mimicking the intracellular Ca^2+^ increase with ionomycin only induces Cx43 opening without affecting cell migration, since prior astrocyte reactivity is necessary for effective cell migration. Therefore, although important, these signals are not sufficient individually, because they only represent part of the intricate network of pathways required to induce cell polarization and migration (Figure 5).

In DITNC1 astrocytes, we showed that Cx43 relocalizes to a juxta-plasma membrane location after Thy-1 treatment. Considering that the increase in Cx43 surface levels occurs rather rapidly (10 min), the processes that involve vesicle trafficking are likely to be important in Cx43 redistribution. In this context, Thy-1 is known to induce the activation of RhoA GTPase [19,20], and RhoA activation by cytoxic necrotizing factor 1 reportedly induces increase of Cx43 gap junctional communication [21]. Trafficking of Cx43 is rapid, as measured by live cell imaging, and involves vesicle movement from the ER to the Golgi, and then on to the plasma membrane in a cytoskeleton-dependent process [22]. Considering that Cx43 hemichannels distribute laterally in the plasma membrane prior to gap junction plaque formation [22], it is possible that Thy-1 stimulates membrane recycling of Cx43 to form hemichannels, rather than gap junctions, at the plasma membrane. The short-term changes in localization of Cx43 and appearance of the protein within a few minutes at the plasma membrane correlate well with our previous results that showed hemichannel opening and ATP release within 10 min following stimulation [4,5]. In view of these results, it is tempting to speculate that Thy-1, through engagement of the integrin, induces Cx43 recycling or trafficking to the astrocyte plasma membrane. Of note, activation of αvβ3 integrin by ligand or antibody binding could be used as a novel modulator of Cx43 hemichannel activity.

We have previously shown that Thy-1 induced cell migration was prevented by Gap19, a specific hemichannel inhibitor [5]. Our current results indicate that cell polarity, an initial essential step in cell migration that positions the Golgi apparatus in the direction of cell migration [5], also depends on hemichannel opening. Other Cxs, such as Cx30, have been implicated in astrocyte polarization. Similar to our results, Cx30 expression in astrocytes led to abnormalities in cellular polarization, β1 integrin redistribution and astrocyte migration, in a process independent of gap junctions [23]. In cardiomyocytes subjected to stretch, Cx43 is overexpressed and the cells spread and polarize their Golgi apparatus to one of the newly formed poles, which contains patches of Cx43 [24]. Taken together, these observations suggest that Cx43 and other Cxs participate in cellular polarization in different types of cells, and not only in astrocytes. Thus, Cxs might be considered common regulators of cell polarization.

The opening of Cx43 hemichannels allows molecules, such as glutamate, ATP, or fluorescent dyes, to cross the plasma membrane [3,10,13,16]. The release of ATP then increases [Ca^2+^]i by activating P2X7R [5,8,11]. Thy-1 induces [Ca^2+^]i increase in astrocytes by releasing Ca^2+^ from intracellular stores and by Ca^2+^ uptake through opening of P2X7R [5,8,11]. In addition, Ca^2+^ signals are required for Cx43 hemichannel opening, and both events are deemed important for polarization and migration induced by Thy-1 [4,5] (Figure 5). However, no previous evidence has indicated if these signals suffice to induce astrocyte migration. We used the Ca^2+^ ionophore ionomycin to generate large [Ca^2+^]i increases. Migration stimulated by Thy-1 in ionomycin-treated cells was lower than that induced by Thy-1 in TNF-treated cells, suggesting that the increase of [Ca^2+^]i alone, is not sufficient to trigger Thy-1-induced cell migration. In astrocytomas, glutamate induces cell migration by regulating Ca^2+^ signals, and the addition of ionomycin leads to glutamate release [17], suggesting a positive feedback loop between [Ca^2+^]i, glutamate release and cell migration. However, in our experiments, migration induced by Thy-1/ionomycin was notably inferior to that induced by TNF/Thy-1, suggesting that astrocyte reactivity and the changes involved in this process require more than just changes in [Ca^2+^]i. Other studies in astrocytes have reported that ionomycin induces NFAT translocation to the nucleus, which is a hallmark of TNF-induced reactive astrocytes [25]; however, nuclear NFAT induced by ionomycin does not activate transcription, unless phorbol 12-myristate 13-acetate, an AP1 and transcriptional co-activator, is included [26]. This is in agreement with our observations, showing that ionomycin alone did not induce migration as well as TNF/Thy-1. When astrocytes were treated with TNF and then with ionomycin, the migration increased to levels similar to those observed for TNF/Thy-1. Our data indicate that TNF induces astrocyte reactivity and the overexpression of several proteins, including β3 integrin, Syndecan-4, P2X7R and Cx43 [4]. Thus, the reduced effect of ionomycin on cell migration in the absence of TNF might be explained by lower endogenous levels of Cx43 and P2X7R, which are not sufficient to generate the positive Ca^2+^ signaling feedback loop, as occurs in a proinflammatory environment [3].

Despite the fact that migration induced by Thy-1/ionomycin is lower in non TNF-treated astrocytes (Figure 4), ionomycin enhances [Ca^2+^]i, but alone, does not suffice to stimulate migration as well as TNF/Thy-1 treatment, suggesting that [Ca^2+^]i induced by Thy-1 requires specific timing or likely occurs in specific domains. As stated above, ionomycin treatment triggers NFAT translocation to the nucleus, but requires phorbol 12-myristate 13-acetate to induce transcription [26]. In addition, the calmodulin-dependent phosphatase calcineurin needs to be present in Ca^2+^ nanodomains, along with Orai1, to induce translocation of NFAT to the nucleus [27], suggesting that a bulk [Ca^2+^]i increase is not sufficient to trigger the complete cellular response. In the same context, the microclusters of β3 integrin formed by integrin overexpression or TNF addition (Figure 2 and Figure 3) may reflect the presence of specific Ca^2+^ nanodomains that activate Ca^2+^ sensors—such as calmodulin/calcineurin—and transcription factors that target proteins involved in cell migration, such as Cx43. However, whether these β3 integrin clusters relate to Ca^2+^ nanodomains remains to be determined.

Reportedly, Thy-1 has two cell surface receptors in astrocytes; however, the main Thy-1 receptor in these cells is β3 integrin [4,5,8]. The second Thy-1 receptor described by our group is Syndecan-4, which binds to Thy-1 with lower affinity than β3 integrin [28], confirming that the key contribution of Thy-1-induced signaling stems from β3 integrin engagement. Integrins are one of the most representative extracellular matrix binding proteins, and upon binding, their clustering is induced rapidly, as determined by other studies that used Single-cell force spectroscopy [29]. These clusters should be formed by freely distributed integrin in the plasma membrane, suggesting that the overexpression of β3 integrin could induce its clustering just by the law of mass action. β3 integrin clustering is a dynamic process that depends on phosphoinositol-4,5-bisphosphate (PI(4,5)P_2_), which functions as a scaffold for integrin docking [30]. Here, we found that both TNF and β3 integrin overexpression induce β3 integrin clustering, suggesting that increased expression of β3 integrin favors Thy-1 binding and/or Thy-1 induced-signaling by receptor clustering (Figure 5).

In summary, we describe here that Cx43 is a key regulator of astrocyte reactivity, polarization and migration. Cx43 hemichannels rapidly move to the plasma membrane by the addition of Thy-1, where they can regulate the release of molecules relevant for astrocyte reactivity, such as ATP. In this study, we show that the [Ca^2+^]i increase required for astrocyte migration can be partially replicated by the addition of ionomycin. However, Thy-1-induced migration in this case is lower than that observed with Thy-1 in TNF-pretreated astrocytes, an effect that relies on the formation of β3 integrin clusters at the plasma membrane, which are not related to focal adhesions, but rather exist in astrocytes that acquire the reactive phenotype.

## 4. Materials and Methods

### 4.1. Cell Line and Primary Cultures

The rat astrocytic cell line DITNC1 (ATCC CRL-2005) was maintained in RPMI medium 1640 (GIBCO, Pittsburgh, PA, USA) containing 5% fetal bovine serum (FBS, HyClone, Pittsburgh, PA, USA), 0.1 mM 2-mercaptoethanol (GIBCO) and 100 U/mL penicillin/100 µg/mL streptomycin (PS mixture, GIBCO). Primary astrocytes were obtained from 1–2-day-old wild-type rats (Wistar strain), as previously described [4]. Cultures were maintained in DMEM/F12 nutrient mixture (1:1), supplemented with 10% FBS and 1% penicillin/streptomycin. To induce a pro-inflammatory environment, rat primary astrocytes were stimulated with TNF 10 ng/mL for 48 h. Both DITNC1 and primary astrocytes were maintained in culture at 37 °C in a 5% CO_2_ humidified atmosphere. The media were changed twice a week and passages were carried out by detaching the cells with 0.1% trypsin (Invitrogen, Grand Island, NY, USA).

### 4.2. Plasmids and Transfections

Astrocytes were transfected with pEGFP-β3 Integrin (full length β3 Integrin subunit kindly donated by Dr. C. Rüegg, University of Friboug, Friboug, Switzerland [31]) or empty vector pEGFP, using the Amaxa Nucleofector system, following the manufacturer’s instructions for the VCA-1003 transfection kit (Lonza, Cologne, Germany).

### 4.3. Fusion Proteins

Thy-1-Fc and Trail-R2-Fc fusion proteins were obtained as described previously [20,32,33]. These fusion proteins were incubated with Protein A (Sigma-Aldrich, St. Louis, MO, USA) in a 10:1 ratio for 1 h at 4 °C prior to their use. Of note, Trail-R2-Fc is a fusion protein of the receptor for the soluble apoptosis-inducing ligand, Trail [34], and is used in these assays as a negative control.

### 4.4. Western Blot

Protein extracts were prepared in a lysis buffer (150 mM NaCl, 0.1% SDS, 0.25% sodium deoxycholate, 1% Triton-X100, in 50 mM Tris-HCl pH 7.4) supplemented with a protease and phosphatase inhibitor cocktail (Biotool, Houston, TX, USA). Extracts were electrophoretically separated on 10% SDS-PAGE gels and transferred to nitrocellulose membranes (Millipore, Billerica, MA, USA), which were blocked with 5% *w*/*v* nonfat, dry milk in PBS containing 0.1% Tween-20, and subsequently incubated with anti-β3 Integrin (Millipore, Billerica, MA, USA) or anti β-actin (Sigma-Aldrich) primary antibodies. The membrane was then washed and incubated with horseradish peroxidase-conjugated goat anti-rabbit IgG (Jackson ImmunoResearch Labs, Inc., West Grove, PA, USA) or goat anti-mouse IgG polyclonal antibody (Bio-Rad Laboratories, Inc., Hercules, CA, USA) for 1 h at room temperature. Bands were visualized with a chemiluminescence kit (Pierce, Thermo Scientific, Rockford, IL, USA), according to the manufacturer’s instructions.

### 4.5. Indirect Immunofluorescence

DITNC1 cells or primary astrocytes were seeded on 12-mm coverslips and left to adhere for 24 h. Cells were then washed and fixed, as previously described [5]. Cells were then stained with anti-Connexin-43 antibody (Santa Cruz Biotechnology, Dallas, TX, USA), followed by secondary antibody conjugated to IF488 (Abbexa, Cambridge, UK). Astrocytes were stained with anti-αvβ3 integrin antibody (Santa Cruz Biotechnology, Dallas, TX, USA) followed by goat anti-mouse IgG conjugated to Alexa fluor 546 (Molecular Probes, Eugene, OR, USA); and DAPI (diamidino-2-phenylindole) (Sigma-Aldrich). Samples were visualized using a confocal Nikon Spectral C2^+^ Plus microscope (Nikon, Tokyo, Japan).

### 4.6. Dye Uptake

Primary astrocytes were seeded on 25-mm coverslips. After 48 h of incubation in complete medium, with or without TNF (10 ng/mL), cells were starved for 30 min and stimulated or not with a complex of Thy-1-Fc-Protein A, TRAIL-R2-Fc-Protein A or ionomicyn (1 µM), for 10 min. Alternatively, dye uptake was evaluated after treating the cells with the hemichannel-blocking peptide Gap19 (Tocris Bioscence, Bristol, UK) (100 μM), scrambled peptide (Tocris Bioscence, Bristol, UK) (300 μM) or BAPTA-AM (5 μM), added 30 min prior to stimulation. After stimulation, cells were incubated at 37 °C with 0.5 mg/mL Lucifer yellow (Sigma-Aldrich) or 2 mg/mL Propidium Iodide (Thermo Fisher Scientific, Waltham, MA, USA) in Ringer Solution (155 mM NaCl, 4.5 mM KCl, 2 mM CaCl_2_, 1 mM MgCl, 10 mM glucose, and 5 mM Hepes, pH 7.4) for 10 min, and then washed twice with the same solution. Fluorescence intensity was quantified in 100 cells per condition in relative units, using the region of interest (ROI) plug-in of the ImageJ software (National Institutes of Health, Bethesda, MD, USA, available online: http://imagej.nih.gov/ij/). Lucifer yellow was excited at 458 nm, and the emission between 500 and 530 nm was quantified. Propidium Iodide was excited at 535 nm, and emission at 617 nm was quantified.

### 4.7. Cell Polarity Assay

Confluent DITNC1 cell monolayers, grown on 12 mm coverslips in 24-well plates, were prepared for wound-healing assays, and then stimulated with Protein A-conjugated Thy-1-Fc or Trail-R2-Fc for 7 h. For experiments with inhibitors, cells were pretreated for 30 min with 100 μM Gap19 (Tocris Bioscience, Bristol, UK). Astrocytes were then fixed and stained as previously described [5]. Coverslips were mounted on slides with 10% Mowiol-2.5% 1,4-Diazabicyclo [2.2.2] octane and samples were observed by confocal microscopy. Cells were considered polarized when the Golgi apparatus was located in the perinuclear area and oriented towards the cell-free zone within a 120° angle (see scheme in Figure 1C). One hundred cells were monitored per condition, and cell polarization was evaluated as the percentage of cells along the wound border exhibiting polarized Golgi structures.

### 4.8. Transwell Migration Assay

Assays were performed in Boyden Chambers (Transwell Costar, 6.5 mm diameter, 8 µm pore size), according to the manufacturer’s instructions. Briefly, the bottom sides of the inserts were coated with 2 µg/mL fibronectin. Cells (5 × 10^4^) in serum-free medium were plated onto the top of each chamber insert and serum-free medium was added to the bottom chamber. After 2 h, inserts were removed, washed, and cells that migrated to the bottom side of the inserts were stained with 0.1% crystal violet in 2% ethanol and counted in an inverted microscope, as previously reported [35,36,37].

### 4.9. Cell Cytometry

Cells were detached using trypsin/EDTA and incubated at 4 °C to avoid internalization of surface proteins. After blocking with BSA 5%, cells were immune-labeled with anti-β3 integrin (Millipore, Billerica, MA, USA) for 60 min. Cells were then washed and incubated with the anti-rabbit Alexa 488 secondary (Molecular Probes) for 30 min. Both incubations were at 4 °C. Cells were analyzed using a FACS Canto (BD Bioscience, San Jose, CA, USA) flow cytometer. Data were analyzed and plotted using FlowJo software (version v10.0.7, Stanford, CA, USA).

### 4.10. Cluster Formation and Confocal Microscopy

Primary astrocytes were seeded on poly-D-lysine coated 25-mm coverslips. The cells were transfected 24 h later with 1 μg of each of the plasmids encoding GFP, GFP-GPI or β3 integrin-GFP for 4 h using Lipofectamine 2000 (Thermo Fisher Scientific), according to manufacturer’s instructions. After 24 h, cells were treated with TNF (10 ng/mL) in complete medium for 2 h, fixed with 4% PFA at 35 °C for 8 min, washed with 1X PBS, and mounted using 10% Mowiol-2.5% 1,4-Diazabicyclo [2.2.2] octane. Samples were photographed using an oil-immersion (HCXAPO_63x/1.40 or 100x/1.40) objective, coupled to a TCS SP5 confocal microscope under sequential scanning mode, and a 2.0-fold digital magnification. Files were digitalized in a 1024 × 1024 pixels format file.

### 4.11. Statistical Analysis

The results are shown as the means ± standard error of the mean (s.e.m.) for *n* = 3 or more experiments as indicated in each figure legend. The results were analyzed using one-way ANOVA tests and Tukey post-tests. Statistical significance was set at *p* < 0.05.

## Abbreviations

TNF	Tumor necrosis factor
Cx43	Connexin 43
IP_3_R	Inositol 1,4,5-trisphosphate receptor
LY	Lucifer yellow
Ca^2+^	Calcium
[Ca^2+^]i	Intracellular Ca^2+^ concentration
CNS	Central nervous system
Px	Pannexin
